# A Neutral Polysaccharide from Ginseng Berry Mitigates D-Galactose-Induced Oxidative Stress and Cognitive Deficits Through the Keap1/Nrf2/HO-1/NQO1 Pathway

**DOI:** 10.3390/antiox15010065

**Published:** 2026-01-03

**Authors:** Ting Ren, Lina Wang, Jiaxin Zhang, Ruitong Song, Xin Li, Jiayue Gao, Xin Sun, Lili Jiao

**Affiliations:** 1 School of Pharmaceutical Sciences, Jilin Medical University, Jilin 132013, China; wlngyf1978@163.com (L.W.); 15144284639@163.com (J.Z.); 13244388229@163.com (R.S.); 13474764716@163.com (X.L.); 18847780456@163.com (J.G.); sunxin@jlmu.edu.cn (X.S.); 2 Jilin Ginseng Academy, Changchun University of Chinese Medicine, Changchun 130117, China

**Keywords:** ginseng berry polysaccharide, structural characteristics, oxidative stress, D-galactose-induced aging-like mice, cognitive deficits

## Abstract

Oxidative stress contributes to brain aging processes and is implicated in related functional decline. Developing strategies to mitigate oxidative stress is therefore of significant interest. In this study, a neutral polysaccharide (GBPN) was isolated from ginseng berry. Structural analysis revealed that GBPN (molecular weight 1.52 × 10^4^ Da) is primarily composed of glucose (53.18%), arabinose (24.3%), and galactose (16.75%). Glucose exists in the forms of →4)-Glc*p*-(1→ (32.95%), →6)-Glc*p*-(1→ (13.81%), and →4,6)-Glc*p-*(1→ (3.70%), while arabinose exists as →1)-Ara*f* (9.73%), →1)-Ara*p* (5.82%), →2)-Ara*p*-(1→ (0.66%), →5)-Ara*f*-(1→ (7.62%), and →3,5)-Ara*f*-(1→ (1.69%) forms, while galactose exists in the forms of →1)-Gal*p* (3.58%), →3)-Gal*p*-(1→ (1.59%), and →3,6)-Gal*p*-(1→ (12.67%). GBPN adopts a triple-helix conformation and exhibits a curled lamellar appearance. Functionally, GBPN exhibited strong 2,2-diphenyl-1-picrylhydrazyl radical, hydroxyl radical scavenging activity, and iron ion chelation capacity. It can activate the antioxidant system in D-galactose-induced aging-like mice, and simultaneously enhance their learning and memory abilities. Mechanistic analysis revealed that these effects are associated with the kelch-like ECH-associated protein 1/nuclear factor erythroid 2-related factor 2 pathway. These findings suggest that ginseng berry polysaccharides like GBPN hold promise as potential agents for alleviating oxidative stress and cognitive deficits in aging-related contexts.

## 1. Introduction

With the increasing trend of aging, population aging has become a common challenge globally, and it is expected that the total number of people aged 60 years and over will reach 1.4 billion in 2030, which will account for one-fifth of the world’s total population [1]. Therefore, healthy aging is related to the well-being of people’s lives globally. Aging involves changes at the level of cells, tissues, organs, and the overall body, and is a multifactorial process that can lead to a wide range of diseases, such as cardiovascular and cerebrovascular diseases, metabolic diseases, cancers, and neurodegenerative diseases [2]. Aging includes age-induced senescence and premature aging caused by stressful stimuli. Physiological senescence occurs with the depletion of the proliferative lifespan over time and is associated with severe shortening of chromosome telomeres. In contrast, stress-induced premature aging is caused by external stimuli such as mitogens, DNA damage, or mitochondrial dysfunction, which can activate aging-related pathways and lead to organismal dysfunction [3,4]. Brain aging and neurodegenerative diseases represent major age-related health challenges. However, it is important to distinguish between natural brain aging processes and specific pathological conditions such as Alzheimer’s disease or Parkinson’s disease. Currently, many interventions targeting age-related cognitive decline or neurodegenerative diseases have limited efficacy and significant side effects [5]. Therefore, exploring safe and effective bioactive compounds from natural sources for mitigating aging-related physiological decline is an important research direction.

Polysaccharides are complex polymers formed by multiple monosaccharide molecules linked by glycosidic bonds, which are distributed across plants, animals, and microorganisms, and serve as essential substrates for maintaining normal physiological functions in living organisms. They have been widely studied because of their good bioactivity, low toxicity and side effects [6]. Polysaccharides have a wide range of biological functions and are involved in cell division and differentiation, regulation of cell growth and senescence, and modulation of immune functions [7,8]. Polysaccharides usually consist of a mixture of polysaccharides with different molecular weights and a mixture of neutral and acidic polysaccharides. Plant-derived neutral polysaccharides have been shown to have good anti-aging effects on the brain. Li et al. found that neutral heteropolysaccharide (RAMP) isolated from *Atractylodes macrocephala* ameliorated D-galactose (D-gal)-induced brain damage and attenuated cognitive decline and oxidative stress in aging mice. It consists of fructose and glucose (Glc) in a molar ratio of 20:1 with a linear backbone of a β-D-(2→1)-linkage [9]. Another neutral polysaccharide, PH-PS, extracted from *Pseudostellaria heterophylla* with a composition of Glc, galactose (Gal) and arabinose (Ara) in a molar mass ratio of 57.78%:41.52%:0.70%, has been shown to ameliorate learning and spatial memory impairments, diminish amyloid β accumulation, and inhibit reactive glia and astrocytes in 5 × FAD mice [10].

Ginseng berry is the mature berry of *Panax ginseng* C. A. Mey. As a renowned traditional Chinese medicinal herb, ginseng possesses calming and cognitive-enhancing properties while delaying aging, which was first documented in the pharmacology compendium Shen Nong’s Classic of Materia Medica [11]. Modern research confirms its role in improving learning and memory capabilities [12]. Based on the principles of holistic perspective, the berry of ginseng is also utilized for cognitive enhancement and anti-aging purposes. Compared to the roots and rhizomes—the traditionally recognized medicinal parts of ginseng—research on ginseng berry remains limited [13]. It has been shown that ginseng berries are rich in polysaccharide components, which have antioxidant effects [14] and may exert anti-immunosenescence effects by inhibiting thymic involution and modulating multiple immune cells that are critical for active immune responses in aged mice [15]. The structure of polysaccharides is complex, and the structural features are closely related to their activities. It is unclear which component of ginseng berry polysaccharides exerts anti-aging effects, and the structural features of ginseng berry polysaccharides have not been resolved in the above studies.

In the present study, we extracted a neutral polysaccharide from ginseng berry with good in vitro antioxidant activity, and investigated its biological effects using the D-gal-induced aging-like mouse model, which is a well-established model for studying accelerated aging phenotypes and aging-related physiological decline. Through structural analysis and in vivo evaluation, we aimed to elucidate the potential protective mechanisms of this polysaccharide, thereby providing a basis for further research on its application in mitigating aging-related dysfunction.

## 2. Materials and Methods

### 2.1. Materials and Chemicals

Five-year-old ginseng berry (GB) was purchased from Ji’an city, China, in October 2023. The samples were identified by Professor Lili Jiao of Changchun University of Chinese Medicine.

DEAE-cellulose and Sepharose CL-6B were the products of Whatman Co. (Maidstone, Kent, UK) and Amersham Pharmacia Co. (Uppsala, Sweden), respectively. Sigma-Aldrich Co. (St. Louis, MO, USA) supplied D-gal, all monosaccharide standards (D-mannose, L-rhamnose, D-glucuronic acid, D-galacturonic acid, D-glucose, D-gal, D-xylose, L-arabinose, and L-fucose), in addition to the reagents 2,2-diphenyl-1-picrylhydrazyl (DPPH, ≥90%), ascorbic acid (vitamin C, ≥99%), EDTA (≥99% purity) and 1-phenyl-3-methyl-5-pyrazolone (PMP). Commercial kits from the Jiancheng Bioengineering Institute (Nanjing, China) were used to determine the activities of malondialdehyde (MDA), superoxide dismutase (SOD), catalase (CAT), glutathione peroxidase (GSH-Px), and total antioxidant capacity (T-AOC). All remaining chemicals and reagents were analytical grade.

### 2.2. Extraction, Separation, and Purification of GBPN

GB (500 g) was extracted with hot water at a solid–liquid ratio of 1:15 for 2 h. The extract was filtered through a 120-mesh nylon cloth, the filtrate was collected, and the residue was extracted twice by repeating the extraction as described above. The three extracts were combined, concentrated to 1000 mL under reduced pressure, alcohol precipitated with 4 times the volume of 95% ethanol, and freeze-dried to obtain ginseng berry crude polysaccharide (GBP). Afterwards, GBP was dissolved in deionized water (10%), and the water-soluble components were further separated and purified using DEAE-cellulose ion-exchange column chromatography and Sepharose CL-6B gel column chromatography, and finally, ginseng berry neutral polysaccharide (GBPN) was obtained.

### 2.3. Analysis of the Structural Characterization of GBPN

#### 2.3.1. Determination of Homogeneity and Molecular Weight of GBPN

The homogeneity of GBPN was determined by high-performance gel permeation chromatography (HPGPC). The chromatographic conditions were: TSKgel G3000 PWxL (Tosoh Bioscience, Shunan-shi, Japan) column (7.8 mm × 30.0 cm), RID-20A detector, the eluent was 0.2 mol/L NaCl, the flow rate of the mobile phase was 0.5 mL/min, the column temperature was 40 °C, and the sampling volume was 20 μL.

The molecular weight of GBPN was analyzed following a previously established protocol [16]. The analysis was conducted on a size exclusion chromatography-multi-angle laser light scattering (SEC-MALLS) system, which comprised an Agilent 1260 (Agilent, Santa Clara, CA, USA) Infinity II high-performance liquid chromatography (HPLC) unit equipped with an OHpak SB-803HQ column (8.0 mm × 30.0 cm, Shodex, Tokyo, Japan). 0.2 mol/L NaCl solution was used as the mobile phase, flowing at 0.6 mL/min, with the column temperature maintained at 35 °C.

#### 2.3.2. Monosaccharide Component Analysis of GBPN

The monosaccharide component of GBPN was analyzed using PMP pre-column derivatization and an HPLC system. In brief, 4 mg of GBPN were hydrolyzed with a methanol solution containing 2 mol/L HCl at 80 °C for 16 h. The reaction solution was blown dry using nitrogen gas, and 0.5 mL of 2 mol/L TFA solution was added for further hydrolysis at 120 °C for 2 h. After hydrolysis was completed, ethanol was repeatedly added to the reaction solution to a pH of 7.0, and the solution was blown dry and left in a vacuum drying oven overnight. PMP derivatization was then carried out according to the method of Ji et al. [17]. The final product was analyzed using an UltiMate 3000 HPLC system (Thermo Finnigan, San Jose, CA, USA) equipped with an Inertsil ODS-3 column (4.6 mm × 150 mm, 5 μm; GL Sciences, Tokyo, Japan). The column was eluted with 82.0% PBS (0.1 mol/L, pH 7.0) and 18.0% acetonitrile at a flow rate of 1.0 mL/min, and the column temperature was set to 25 °C.

#### 2.3.3. Fourier-Transform Infrared Spectroscopy (FT-IR) Analysis of GBPN

Thoroughly mix 2.0 mg of completely dried GBPN with 200 mg of KBr powder. Grind the mixture and press it into a 1.0 mm pellet, which is then measured using a Thermo Scientific Nicolet iS-5 spectrometer (Thermo Finnigan, San Jose, CA, USA). The spectral recording covers the wavenumber range of 400–4000 cm^−1^.

#### 2.3.4. Methylation and Gas Chromatography-Mass Spectrometry Analysis of GBPN

A previously reported protocol was employed, in which GBPN was subjected to methylation, hydrolysis, reduction, and finally acetylation [18]. Then, the obtained partially methylated alditol acetates (PMAAs) were detected via GC–MS by using a TSQ 8000 instrument (Thermo Fisher Scientific, Waltham, MA, USA) equipped with a TG-5SiLMS column (30 m × 0.25 mm × 0.25 μm). The column temperature protocol commenced with a 2 min hold at 120 °C, and then increased to 280 °C for 5 min at a rate of 10 °C/min and finally held for 2 min. A helium carrier gas was used with a flow rate of 1.0 mL/min.

#### 2.3.5. Scanning Electron Microscope (SEM) Observation

The surface morphology and microstructure of GBPN were examined using an SEM (Hitachi S-3000N, Hitachi, Tokyo, Japan). Following a standard preparation procedure, the GBPN powder was sputter-coated with a thin gold film under vacuum conditions and then tested at an acceleration voltage of 30.0 kV.

#### 2.3.6. Conformational Characteristics Analysis

The Congo red experiment was applied to investigate the conformational characteristics of GBPN. Briefly, 2 mg/mL polysaccharide solution (2 mL) was combined with an equal volume of 91 μmol/L Congo red reagent. Subsequently, NaOH was introduced into the mixture to achieve final concentrations ranging from 0 to 0.7 mol/L. Congo red solution without the polysaccharide served as the control. Absorbance values across 400–700 nm were recorded. A curve was then generated by plotting the maximum absorption wavelength against the corresponding NaOH concentration.

### 2.4. Determination of Antioxidant Capacity In Vitro

#### 2.4.1. Assay for DPPH Radical Scavenging Activity

The DPPH radical scavenging ability of GBPN was determined by the method of literature [19]. 100 µL of 0.01–1.5 mg/mL of GBPN and vitamin C were taken, respectively, and 100 µL of 2 × 10^−4^ mol/L DPPH solution (prepared with anhydrous ethanol) was added, mixed well, and then left for 30 min at room temperature (avoiding light). The absorbance was measured at 517 nm, and the clearance rate calculated according to the following formula:

(1) DPPH scavenging rate (%) = [1 − (A3 − A2)/(A1 − A0)] × 100%,

where A3 was the absorbance of different concentrations of sample plus DPPH; A2 was the absorbance of different concentrations of sample plus distilled water; A1 was the absorbance of distilled water plus DPPH; and A0 was the absorbance of distilled water.

Each concentration was tested in triplicate, and the entire experiment was independently repeated three times (*n* = 3).

#### 2.4.2. Assay for Hydroxyl Radical (^•^OH) Scavenging Activity

Detection of ^•^OH scavenging activity using the Fenton reaction [20]. Briefly, 50 μL of different concentrations of GBPN solution and vitamin C solution (0.125–2 mg/mL) were mixed with 50 μL of H_2_O_2_ (0.025%, *w*/*v*), 50 μL of sodium salicylate (9.0 mmol/L), and 50 μL of FeSO_4_ (9.0 mmol /L) thoroughly, the absorbance Ai was measured at 562 nm after the reaction at 37 °C for 30 min, where vitamin C solution was used as a positive control. The absorbance measured with distilled water instead of polysaccharide solution was recorded as A0, and the absorbance measured with different concentrations of polysaccharide solution plus distilled water solution as a colour control was recorded as Aj.

The scavenging rate of ^•^OH was calculated according to the following formula:

(2) ^•^OH scavenging rate (%) = [1 − (Ai − Aj)/A0] × 100%,

Measurements for all concentrations were performed in triplicate, and the complete experiment was replicated three times independently (*n* = 3).

#### 2.4.3. Assay for Ferrous Ion Chelating Ability

Referring to the method of Wang et al. [21], 100 μL of GBPN and EDTA solutions with different concentrations (0.25–1.5 mg/mL) were incubated with 50 μL of FeSO_4_ solution (0.125 mM) and 50 μL of phenoxazine (1 mM) at 37 °C for 10 min. The absorbance at 562 nm was determined. The formula is as follows:

(3) Ferrous ion chelating ability (%) = [1 − (A1 − A2)/A0] × 100%,

where A0 is the absorbance measured by distilled water instead of the sample, A1 is the absorbance measured by the sample solution, and A2 is the absorbance measured by the sample solution plus distilled water.

All assays were conducted in triplicate, and three independent experimental runs were performed (*n* = 3).

### 2.5. Evaluation of In Vivo Antioxidant and Anti-Brain Aging Activities

#### 2.5.1. Animals and Treatment

The animal study utilized 36 male Kunming mice (6 weeks old, 30 ± 2 g) supplied by the Experimental Animal Centre of Jilin University. The use of male mice was based on the following considerations: (1) to maintain consistency with previous studies using the D-gal-induced aging-like model in mice, and (2) to minimize variability due to sex hormones in this preliminary mechanistic investigation. Mice were housed in groups of six per cage under standard laboratory conditions: a 12 h/12 h light/dark cycle, a temperature of 21 °C ± 2 °C, and 55% ± 5% relative humidity. These mice had free access to food and water.

After one week of adaptive feeding, mice were grouped using a random number table method. The procedure was as follows: 36 mice were numbered in ascending order of body weight. Using a computer-generated random number sequence, mice were evenly distributed into the following 6 groups (n = 6 per group): control, negative, vitamin C, GBPN-50, GBPN-100, and GBPN-200. Randomization was performed by researchers not involved in subsequent behavioral and biochemical testing to ensure objectivity. Following grouping, all groups except the control group established an aging-like model via intraperitoneal injection of D-gal (prepared in physiological saline at a concentration of 135 mg/mL to achieve a dosage of 1.35 g/kg/d, with injection volume adjusted according to the body weight of each mouse; the dose was determined by pre-experimentation). At the same time, vitamin C group mice were given 100 mg/kg/d of vitamin C by gavage, mice in the GBPN groups were given GBPN by gavage at doses of 50, 100 and 200 mg/kg/d, respectively (GBPN dosage determined by preliminary experiments). All groups were given the D-gal, vitamin C or GBPN for forty-two consecutive days. The body weight of the mice was measured every 7 days, and the administered dose was adjusted accordingly. The mice were sacrificed 12 h after the last drug administration. Then, serum (centrifuged at 3000× *g* for 10 min), liver, and brain were collected. This animal study protocol was approved by the Ethics Committee of Laboratory Animals of Jilin Medical University (Protocol No.: 2024-GKJJ-019, Approval Date: 11 March 2024).

#### 2.5.2. Analysis of Oxidative Stress Indicators

The levels of SOD, CAT, GSH-Px, T-AOC and MDA in serum, liver and brain of mice were determined using commercial assay kits (Nanjing Jiancheng Bioengineering Institute, Nanjing, China) according to the manufacturer’s instructions. Specifically, the kits used were as follows: SOD assay kit (Catalogue No. A001-3-2), CAT assay kit (Catalogue No. A007-1-1), GSH-Px assay kit (Catalogue No. A005-1-2), T-AOC assay kit (Catalogue No. A015-2-1), and MDA assay kit (Catalogue No. A003-1-2). Briefly, serum samples were obtained by centrifugation, while liver and brain tissues were homogenized in ice-cold physiological saline (1:9, *w*/*v*) and centrifuged to collect the supernatant. The protein concentration of tissue homogenates was quantified using a BCA protein assay kit. For each assay, appropriate volumes of sample, working reagents, and standards were mixed and incubated under the specified conditions (temperature and time) as per each kit’s protocol. Absorbance was measured using a microplate reader at the wavelengths indicated in the respective kit manuals. All measurements were performed in duplicate, and the final levels of each oxidative stress marker were calculated based on the corresponding standard curves and normalized to protein concentration for tissue samples.

#### 2.5.3. Morris Water Maze (MWM) Test

The MWM test consisted of a place navigation trial and a spatial exploration trial, conducted with minor modifications based on a previous study [7]. The experiments were performed in a circular pool divided into four quadrants and filled with water (25 ± 1 °C). In the place navigation trial, an escape platform was placed in the north-east quadrant of the pool (1 cm below the water surface), and the mice were trained four times a day for 6 days. Results from the sixth day are presented in this study. Mice were placed into the water from four different quadrants facing the wall of the pool, and the time required for the mice to find and climb up to the platform (escape latency) was recorded. If the mice did not find the platform within 120 s, they had to be guided to the platform and stayed on the platform for 20 s, which was recorded as the escape latency of 120 s. If the mice reach the platform within 120 s, they are allowed to remain on the platform for 10 s, and the time taken to reach the platform is recorded. All trials, including those involving guiding mice onto the platform, were included in the analysis. For statistical analysis, the escape latency for guided trials was consistently recorded as 120 s.

The spatial exploration trial was performed on day 7. The escape platform was removed from the pool, and each mouse was placed in the water in the south-west quadrant and allowed to swim for 120 s. The residence time of the mice in the north-east quadrant of the pool and the number of times they crossed the platform were recorded, while its complete swimming trajectory was documented.

#### 2.5.4. Mechanism Analysis

The protein expression levels of kelch-like ECH-associated protein 1 (Keap1), nuclear factor erythroid 2-related factor 2 (Nrf2), heme oxygenase 1(HO-1), and NAD(P)H: quinone oxidoreductase 1 (NQO1) in the mouse brain tissues were detected using the Western blotting method. Total proteins were extracted by RIPA lysate after tissue homogenization, and protein concentration was quantified by the BCA kit. Proteins were separated using SDS-PAGE electrophoresis and then transferred to polyvinylidene difluoride (PVDF) membranes. After the PVDF membranes were closed, anti-GAPDH antibody (Abcam, Cambridge, UK), anti-Keap1 antibody (Abcam, UK), anti-Nrf2 antibody (Abcam, UK), anti-HO-1 antibody (Abcam, UK), and anti-NQO1 antibody (Abcam, UK) were added and incubated at 4 °C overnight. Following a TBST wash, the PVDF membrane was subjected to a 2 h incubation at room temperature with a horseradish peroxidase-conjugated secondary antibody (Nachuan Biotech, Changchun, China). After another TBST wash, the protein bands were visualized using an NcmECL Ultra detection kit (NCM Biotech, Suzhou, China), and their intensity was quantified with ImageJ software (version 1.54).

### 2.6. Statistical Analysis

All data are presented as the mean ± standard deviation (SD). For comparisons among multiple groups at a single time point, one-way analysis of variance (ANOVA) followed by Tukey’s post hoc test was used. For repeated measurements over time (body weight), a two-way repeated measures ANOVA was applied with Time and Group as factors, followed by Tukey’s test for multiple comparisons when significant interactions or main effects were detected. Mauchly’s test of sphericity was used to assess the homogeneity of covariances. Where the assumption of sphericity was violated (*p* < 0.001), the Greenhouse–Geisser correction was applied to adjust the degrees of freedom for the within-subject factor (Time) and the interaction (Time × Group). Prior to conducting parametric tests, the normality of distribution for each dataset was assessed using the Shapiro–Wilk test, and the homogeneity of variances was verified using Levene’s test. All datasets met the assumptions for ANOVA (*p* > 0.05 for both normality and homogeneity tests, except for the sphericity test in repeated measures). All analyses were performed using IBM SPSS Statistics software, Version 26.0 (IBM Corp., Armonk, NY, USA), with a *p*-value of less than 0.05 considered statistically significant. Data visualization and subsequent processing were performed with Origin 2018 software.

## 3. Results and Discussion

### 3.1. Isolation and Purification of GBPN

**Figure 1:**
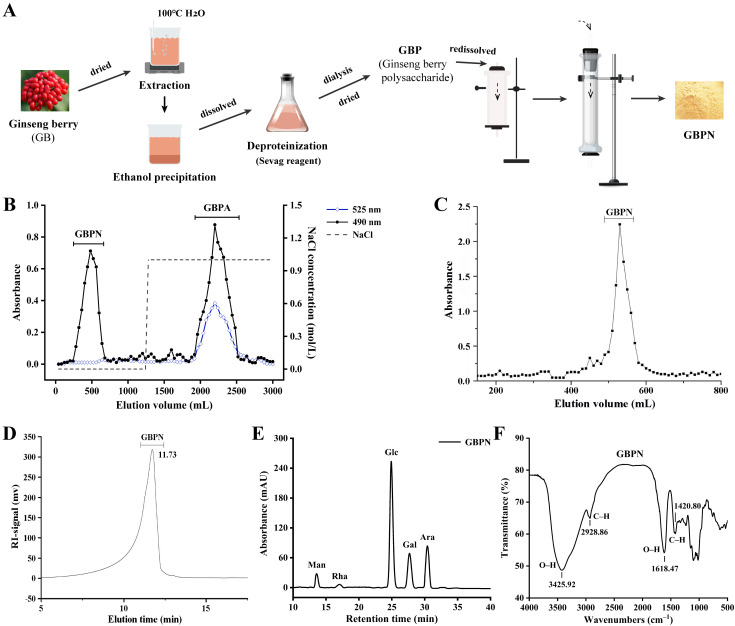
Extraction, isolation, purification and primary structure of GBPN. (**A**) Extraction, isolation and purification chart (The dashed arrows indicate the flow path of the sample); (**B**) DEAE-cellulose elution profile; (**C**) Sepharose CL-6B elution profile (Each square represents the absorbance of polysaccharides in the eluted fractions determined by the phenol-sulfuric acid method. The line is the elution curve plotted based on these data); (**D**) HPLC chromatogram diagram; (**E**) Monosaccharide composition diagram; (**F**) FT-IR spectrum.

Figure 1A shows the whole process of extraction and purification of neutral sugar GBPN from ginseng berries. Ginseng berry crude polysaccharide (GBP) was obtained by aqueous extraction-alcohol precipitation, deproteinization and dialysis, with a yield of 7.12% relative to the raw material. GBP was separated by DEAE-cellulose column to obtain the neutral polysaccharide GBPN (Figure 1B) [16], with a yield of 2.05% relative to the raw material. Subsequently, GBPN was further purified using a Sepharose CL-6B gel column, and the eluent of the main components was dialysed (molecular weight cut-off of 3500 Da) and then freeze-dried to obtain homogeneous GBPN (0.31% yield in the raw material) (Figure 1C). The total sugar content of GBPN was 93.5 ± 7.4%, and the protein content was 5.46 ± 0.68%.

### 3.2. Structural Characterization of GBPN

#### 3.2.1. Primary Structural Characterization

As shown in Figure 1D, the single symmetric peak observed in HPLC indicated that GBPN is a homogeneous polysaccharide. Furthermore, SEC-MALLS results indicated that GBPN has a molecular weight of 1.52 × 10^4^ Da and a polydispersity (Mw/Mn) value of 1.19, consistent with the HPLC results. The monosaccharide composition analysis revealed that GBPN was mainly composed of Glc (53.18%), Ara (24.3%) and Gal (16.75%), with small amounts of mannose (Man 4.55%) and rhamnose (Rha 1.22%) (Figure 1E). Methylation analysis showed that in GBPN, Glc exists in the forms of →4)-Glc*p*-(1→, →6)-Glc*p*-(1→ and →4,6)-Glc*p*-(1→, accounting for 32.95%, 13.81%, and 3.70%, respectively, Ara is present as →1)-Ara*f*, →1)-Ara*p*, →2)-Ara*p*-(1→, →5)-Ara*f*-(1→, and →3,5)-Ara*f*-(1→, accounting for 9.73%, 5.82%, 0.66%, 7.62%, and 1.69%, respectively, Gal is present in the forms of →1)-Gal*p*, →3)-Gal*p*-(1→, and →3,6)-Gal*p*-(1→, accounting for 3.58%, 1.59%, and 12.67%, respectively, Man and Rha are both present in the form of single glycosidic bonds, with →1)-Man*p* accounting for 4.86% and →2,4)-Rha*p*-(1→ accounting for 1.34%, consistent with the results of the monosaccharide composition (Table 1). The total ion gas chromatogram and mass ion fragment diagram for GBPN are shown in Supplementary Figures S1 and S2, respectively. Based on the above results, GBPN may possess a backbone primarily composed of →4)-Glc*p*-(1→ linkages, containing a significant proportion of →6)-Glc*p*-(1→ linkages as potential branching points or linear extensions. Ara residues primarily exist in terminal (→1)-Ara*f*/*p*) and →5)-Ara*f*-(1→ forms, potentially constituting side chains. The →3,6)-Gal*p*-(1→ linkage of Gal residues also suggests the presence of branching structures.

The molecular weight, monosaccharide composition and glycosidic linkage patterns of GBPN differed from the neutral polysaccharides of ginseng berries reported in the literature. Feng et al. reported that neutral polysaccharide (AGBP-A) of ginseng berry with a molecular weight of 12.3 × 10^4^ Da is composed of Ara and Gal with a core structure containing →6)-Gal*p*-(1→ residues as the backbone and a branching substitution at the C3 position, and the side chains comprising α-L-Ara*f*-(1→, →5)-α-L-Ara*f*-(1→, β-D-Gal*p*-(1→, exhibited in vitro and in vivo anti-inflammatory activity [22].

Figure 1F shows the FT-IR spectrum of GBPN. The intense bands at 3425.92 cm^−1^ and 1618.47 cm^−1^ are attributed to the presence of O–H stretching vibration in the hydrogen bonds, indicating strong intermolecular interactions between the polysaccharide chains [23,24]. The absorption band at 2928.86 cm^−1^ represents the C–H stretching vibration. In addition, there is a stretching vibration at 1420.80 cm^−1^ caused by C–H. In summary, GBPN is a neutral polysaccharide.

#### 3.2.2. Morphology and Conformation Analysis of GBPN

**Figure 2:**
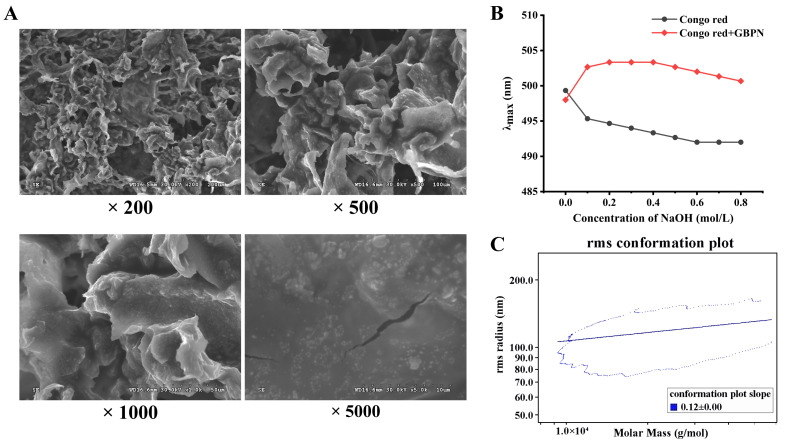
The morphological and conformational features of GBPN. (**A**) SEM photographs, magnification: 200×, 500×, 1000×, 5000×; (**B**) Congo red experiment; (**C**) SEC-MALLS chain conformation analysis.

The apparent structures of polysaccharides are diverse and have an influence on their biological activities [25]. The microstructure of GBPN at different magnifications was observed using SEM (Hitachi, Tokyo, Japan). As shown in Figure 2A, at 200×, GBPN showed irregular curled flakes; at 1000×, GBPN had an unsmooth surface with bumps and no pores; after magnification to 5000×, GBPN was visible as a flaky structure with irregular particles. Polysaccharides with rough flake structure, such as fermented *Ganoderma lucidum* polysaccharides [26] and *Salvia miltiorrhiza* Bunge polysaccharides [27], have antioxidant activity and are particularly significant for the modulation of Keap1 and Nrf2-mediated antioxidant responses, which may be attributed to the fact that the structures on their surfaces expose more active sites and increase the surface area, which helps in utilization and absorption.

The Congo red test is used to determine whether a polysaccharide has a helical structure. Polysaccharides with triple helical structure combine with Congo red to form a complex, and the maximum absorption wavelength (λmax) of the complex will be red-shifted compared to a single Congo red solution. When there is a strong alkali effect, the triple helical conformation of the polysaccharide is destroyed, resulting in a decrease in the red-shift effect of the complex [28]. As shown in Figure 2B, the λmax values of the Congo red-GBPN complexes were red-shifted with increasing NaOH concentration in the range of 0~0.4 mol/L NaOH concentration, which indicates that GBPN has a triple helical structure. The red-shift effect of the complexes gradually decreased with further increase in NaOH concentration. Plant polysaccharides with a triple helical structure are usually reported to have good antioxidant activity, such as *Glycyrrhiza inflata* Batal. polysaccharide [29], *Neolamarckia cadamba* fruits polysaccharides [30], and *Polygonatum rhizome* polysaccharides [31]. GBPN is a plant-derived polysaccharide with triple helix characteristics which has similar antioxidant properties.

The chain conformation of the polysaccharide solution was determined based on the linear slope (α value) of the radius of gyration (Rg) versus molecular molar mass plot in the SEC-MALLS system. The α value of GBPN was 0.12 ± 0.00, indicating that GBPN has a spherical conformation in aqueous solution (Figure 2C). Study reported that molecules with slopes less than or equal to 0.3 have a tight and uniform spherical conformation in solution; those with slopes between 0.5 and 0.6 have a random coil conformation; and those with slopes > 0.6 have an extended structure [32].

In summary, GBPN exhibits significant differences from previously reported neutral polysaccharides from ginseng berry in terms of molecular weight, monosaccharide composition, and glycosidic linkage patterns [22]. The molecular weight of GBPN (15.2 kDa) is substantially lower than that of AGBP-A (123 kDa), which may influence its solubility, absorption rate, and biological activity. Furthermore, AGBP-A consists solely of Ara and Gal, whereas GBPN predominantly features Glc (53.18%) alongside Ara, Gal, and minor amounts of Man and Rha, suggesting distinct biosynthetic origins or extraction specificities. Bond analysis further revealed that GBPN possesses a richer combination of glycosidic bonds, including Glc in the →4)-Glc*p*-(1→, →6)-Glc*p*-(1→, and →4,6)-Glc*p-*(1→ forms, Ara in the →1)-Ara*p*, →2)-Ara*p*-(1→, and →3,5)-Ara*f*-(1→ forms, Gal’s →3)-Gal*p*-(1→ and →3,6)-Gal*p*-(1→, and →1)-Man*p* and →2,4)-Rha*p*-(1→ glycosidic bond forms, which are not reported in AGBP-A. Furthermore, GBPN adopts a triple-helix conformation, whereas the conformation of AGBP-A remains undetermined. These structural distinctions highlight the novelty of GBPN, suggesting it may represent a unique polysaccharide fraction within ginseng berries possessing specific functional properties.

### 3.3. Antioxidant Activities of GBPN In Vitro

#### 3.3.1. DPPH Radical Scavenging Ability

**Figure 3:**
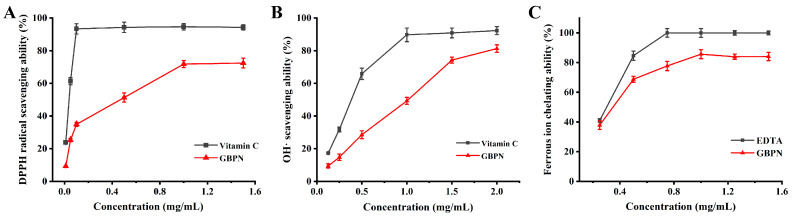
The antioxidant activities of GBPN. (**A**) scavenging DPPH radical activity; (**B**) scavenging ^•^OH activity; (**C**) chelating Fe^2+^ ability. Data are presented as mean ± SD (*n* = 3 independent experiments).

The DPPH method has been used for the determination of antioxidant capacity in vitro since it was proposed in 1958 [33]. As shown in Figure 3A, the DPPH radical scavenging ability of GBPN increased in a dose-dependent manner in the concentration range of 0.01–1.0 mg/mL. When the concentration of GBPN was increased to 1.0 mg/mL, the scavenging ability reached a maximum value of 71.85 ± 2.11% and levelled off, whereas the positive control vitamin C had a stronger scavenging capacity for DPPH radicals than that of GBPN, and its clarity rate reached the maximum value of 93.42 ± 3.17% at 0.10 mg/mL. The IC_50_ values were determined to be approximately 0.31 mg/mL for GBPN and 0.03 mg/mL for vitamin C, further confirming the superior DPPH radical scavenging activity of vitamin C, while still indicating considerable antioxidant potential of GBPN.

#### 3.3.2. ^•^OH Scavenging Ability

^•^OH are reactive oxygen radicals, which can react with biomolecules such as DNA, proteins and lipids in organisms, leading to oxidative damage. Removal of ^•^OH can alleviate the occurrence of oxidative stress and reduce cell and tissue damage [19]. The concentration of GBPN was positively correlated with the ^•^OH scavenging activity in the concentration range of 0.125–2 mg/mL, and its ^•^OH scavenging ability increased from 9.49 ± 1.28% to 81.32 ± 2.29% when the polysaccharide concentration was increased from 0.125 mg/mL to 2 mg/mL (Figure 3B). Meanwhile, the ^•^OH scavenging activity of vitamin C increased from 17.34 ± 0.57% to 92.29 ± 2.47%. The calculated IC_50_ values further quantified this activity, with GBPN showing an IC_50_ of approximately 0.83 mg/mL, compared to approximately 0.35 mg/mL for vitamin C. These results demonstrate that GBPN exhibits potent ^•^OH scavenging activity, highlighting its potential as a natural antioxidant.

#### 3.3.3. Ferrous Ion Chelating Ability

Ferrous ions can participate in the Fenton reaction to generate hydroxyl radicals in vivo. Chelation of ferrous ions by antioxidants may thus suppress free radical formation. In this study, the chelating ability of GBPN on ferrous ions was determined to assess its antioxidant activity. As shown in Figure 3C, the chelating ability of GBPN on ferrous ions is positively correlated with the concentration in the range of 0.25–1.0 mg/mL, and reaches a maximum value of 85.68 ± 2.98% at 1.0 mg/mL, and the chelating ability of EDTA as a positive control is stronger than that of GBPN, and reaches a maximum value of 100% at 0.75 mg/mL. The corresponding IC_50_ values were approximately 0.32 mg/mL for GBPN and 0.28 mg/mL for EDTA. These results indicate that GBPN possesses notable ferrous ion chelating capacity, supporting its relevance as an antioxidant agent.

In the aforementioned in vitro experiments, the antioxidant activity of GBPN was measured at concentrations as high as 1–2 mg/mL. This concentration range was selected to establish a comprehensive dose–response curve and to evaluate GBPN’s intrinsic radical scavenging and metal chelation capabilities under controlled conditions. Subsequent in vivo dosing of GBPN (50–200 mg/kg/d) was determined based on prior efficacy studies and aligns with reported dosages of other plant polysaccharides in aging models [34].

### 3.4. Effect of GBPN on D-Gal Induced Aged Mice

#### 3.4.1. GBPN Improves Body Weight in D-Gal-Induced Aged Mice

**Figure 4:**
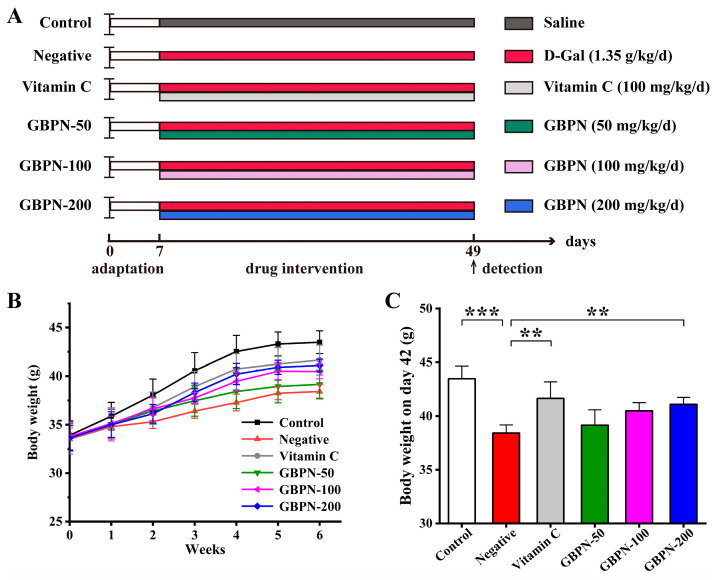
Animal experimental procedures and the effect of GBPN on body weight in D-gal-induced aging-like mice. (**A**) Animal experimental procedure chart; (**B**) Body weight; (**C**) Body weight on day 42. Data are presented as mean ± SD (n = 6). Statistical analysis for body weight over time was performed using two-way repeated measures ANOVA with Tukey’s post hoc test. ** *p* < 0.01, *** *p* < 0.001 vs. Negative group.

This study recorded the mouse body weight during drug administration. As shown in Figure 4B, no significant differences in body weight were observed among the six groups at the start of the experiment. A two-way repeated measures ANOVA revealed a significant main effect of time (F(2.16, 64.83) = 911.61, *p* < 0.001, partial η^2^ = 0.97) and group (F(5, 30) = 5.01, *p* = 0.002, partial η^2^ = 0.46), and a significant time × group interaction (F(10.80, 64.83) = 11.72, *p* < 0.001, partial η^2^ = 0.66) (corrected using Greenhouse–Geisser). This indicates significant differences in weight changes over time among treatment groups. Analysis of body weight at the final time point (day 42) revealed that mice in the Negative group exhibited significantly lower body weight than the Control group following D-gal treatment (*p* < 0.001) (Figure 4C). Compared to the Negative group, body weights in the GBPN groups exhibited dose-dependent increases. The GBPN-200 group showed significantly higher body weights than the Negative group at the end of treatment (*p* < 0.01), though still lower than the Vitamin C group (Figure 4C). Additionally, univariate analyses at each time point revealed statistically significant differences in body weight between the Control and Negative groups starting from week 2 (*p* < 0.05). Beginning at week 4, the GBPN-200 group demonstrated significant differences compared to the Negative group (*p* < 0.05). This indicates that GBPN mitigates D-gal-induced age-related weight loss in mice.

#### 3.4.2. GBPN Downregulated Oxidative Stress Levels in D-Gal-Induced Aging-like Mice

**Figure 5:**
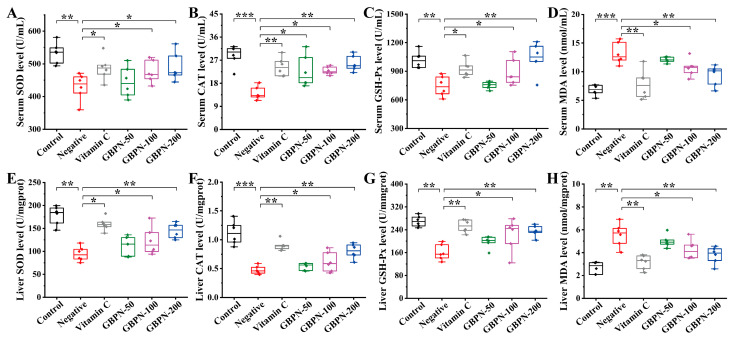
Effects of GBPN on the levels of SOD, CAT, GSH-Px and MDA in the serum and liver of D-gal-induced aging-like mice. (**A**) serum SOD, (**B**) serum CAT, (**C**) serum GSH-Px, (**D**) serum MDA, (**E**) liver SOD, (**F**) liver CAT, (**G**) liver GSH-Px, (**H**) liver MDA. Data are presented as mean ± SD (n = 6). Statistical significance was determined by one-way ANOVA followed by Tukey’s post hoc test. * *p* < 0.05, ** *p* < 0.01, *** *p* < 0.001 vs. Negative group.

The antioxidant capacity of GBPN was assessed by measuring the levels of SOD, CAT, GSH-Px and MDA in serum and liver of mice in each group. Compared with the Control group, mice in the Negative group showed decreased levels of SOD, CAT and GSH-Px and increased levels of MDA (*p* < 0.01 or *p* < 0.001). After 6 weeks of administration, GBPN increased the levels of SOD, CAT and GSH-Px and inhibited the overproduction of MDA in a dose-dependent manner (Figure 5). Compared with the Negative group, GBPN at 200 mg/kg increased serum levels of SOD, CAT and GSH-Px by 14.04%, 82.03% and 39.59% (*p* < 0.05, *p* < 0.01, *p* < 0.01), respectively (Figure 5A–C), and liver levels of SOD, CAT and GSH-Px by 53.69%, 69.25% and 46.63% (*p* < 0.01), respectively (Figure 5E–G). On the contrary, GBPN at 200 mg/kg decreased the levels of MDA in serum and liver by 28.68% and 31.94% (*p* < 0.01), respectively, when compared with the Negative group (Figure 5D,H). The above results suggest that GBPN has a positive effect on oxidative stress in aged mice.

#### 3.4.3. GBPN Ameliorates Spatial Memory Dysfunction and Brain Oxidative Stress Injury in D-Gal-Induced Mice

It has been shown that some plant polysaccharides, such as Angelica polysaccharide, *Polygonatum sibiricum* polysaccharides and *Momordica charantia* polysaccharide, can ameliorate brain senescence and oxidative stress damage in D-gal-induced aged mice, and improve cognitive impairment and spatial learning and memory abilities [8,35,36].

**Figure 6:**
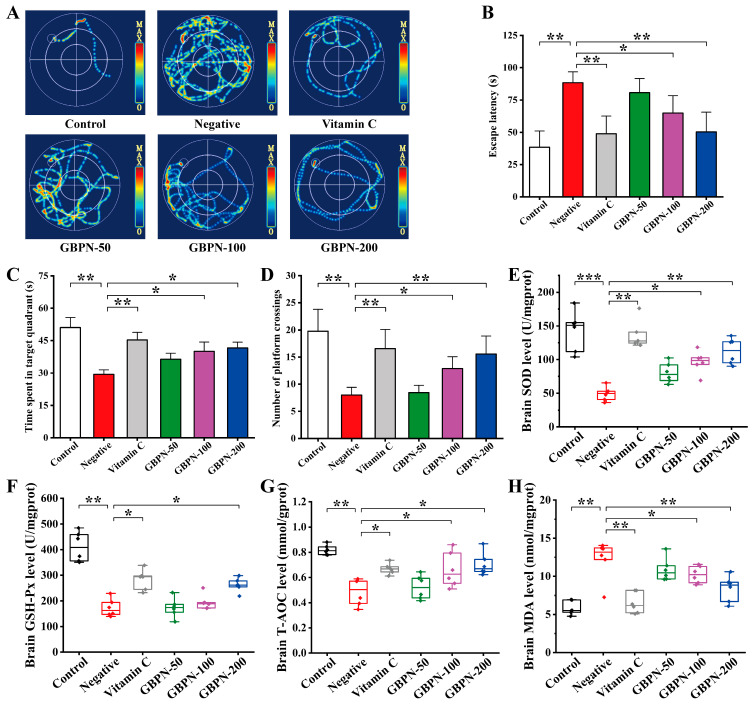
Effects of GBPN on cognitive function and oxidative stress in the brain of D-gal-induced aging-like mice. (**A**) Representative swimming trajectories of experimental mice on the final day, (**B**) Escape latency on day 6 in MWM place navigation trial, (**C**) Time spent in the target quadrant for MWM spatial exploration trial, (**D**) Number of platform crossings for MWM spatial exploration trial, (**E**) brain SOD level, (**F**) brain GSH-Px level, (**G**) brain T-AOC level, (**H**) brain MDA level. Data are presented as mean ± SD (n = 6). Statistical analysis for (**B**–**H**) was performed using one-way ANOVA followed by Tukey’s post hoc test. * *p* < 0.05, ** *p* < 0.01, *** *p* < 0.001 vs. Negative group.

In this study, the effect of GBPN on D-gal-induced cognitive impairment in aged mice was assessed using the MWM experiment. The results of the place navigation trial are shown in Figure 6B. On day 6 of training, the escape latency of mice in the negative group was significantly prolonged compared to the control group (*p* < 0.01). The escape latency of mice in the GBPN group at the dose of 200 mg/kg/d was the shortest and closest to that of the vitamin C group compared to the GBPN groups at the doses of 50 mg/kg/d and 100 mg/kg/d, and it was significant when compared to the negative group (*p* < 0.01). This suggests that GBPN has an ameliorating effect on the prolongation of escape latency in D-gal-induced aged mice. The results of the spatial exploration trial on day 7 are shown in Figure 6C,D. Compared with the control group, the residence time in the target quadrant and the number of crossing platforms in the negative group were significantly reduced, indicating that the spatial learning and memory abilities of mice in the negative group were impaired. Compared with the negative group, the cognitive functions of the mice in the GBPN group showed a gradual improvement trend with the increase in dose, and the residence time in the target quadrant and the number of crossing platforms in the mice of the GBPN group at the dose of 200 mg/kg/d were the closest to that of the vitamin C group, which was significant compared with that of the negative group (*p* < 0.05, *p* < 0.01). The above findings suggest that GBPN can dose-dependently ameliorate D-gal-induced impaired spatial learning and memory abilities in aging-like mice. Representative swim path diagrams (Figure 6A) show typical search patterns without apparent motor abnormalities across groups, supporting that the improvements in escape latency and spatial exploration are likely cognitive rather than motor in origin.

The results of the biochemical assessment in the brain are shown in Figure 6E–H. The MDA content in the brain of mice in the Negative group was significantly higher compared with the control group (*p* < 0.01), and the brain SOD, GSH-Px and T-AOC contents were significantly lower (*p* < 0.01, *p* < 0.001). This indicated that D-gal caused the presence of brain oxidative damage in the mice in the negative group. In contrast, brain MDA content was significantly reduced (*p* < 0.01) (Figure 6H) and brain SOD, GSH-Px and T-AOC were significantly increased (*p* < 0.05, *p* < 0.01) (Figure 6E–G) by GBPN at 200 mg/kg/d compared with the negative group, suggesting that GBPN has a role in resisting D-gal-induced brain oxidative stress injury.

It should be noted that this study primarily evaluated the anti-aging effects of GBPN on brain aging at the biochemical and behavioral levels, without conducting morphological analysis of brain tissue or detecting neuronal damage markers. Therefore, our conclusions primarily support GBPN’s capacity to improve oxidative stress status and cognitive dysfunction in brain aging-like models. Although tissue GBPN concentrations were not measured, the significant improvement in cognitive function and oxidative stress markers in D-gal-induced aged mice indicates that the administered GBPN dose holds pharmacological significance. Future studies should employ systematic histological and neuronal injury marker analyses alongside pharmacokinetic investigations to comprehensively validate GBPN’s neuroprotective effects and determine its bioavailability, tissue distribution, and achievable concentrations in target organs such as the brain.

#### 3.4.4. Association of GBPN with the Keap1/Nrf2/HO-1/NQO1 Pathway in D-Gal-Induced Mice

The Keap1/Nrf2 pathway is a recognized target for the prevention and treatment of oxidative stress-related diseases. Under physiological conditions, Keap1 binds to Nrf2, retaining it in the cytoplasm and maintaining its inactivity. Upon oxidative stress, Nrf2 dissociates from Keap1, translocates to the nucleus, and activates the expression of antioxidant enzymes such as HO-1 and NQO1 [37].

**Figure 7:**
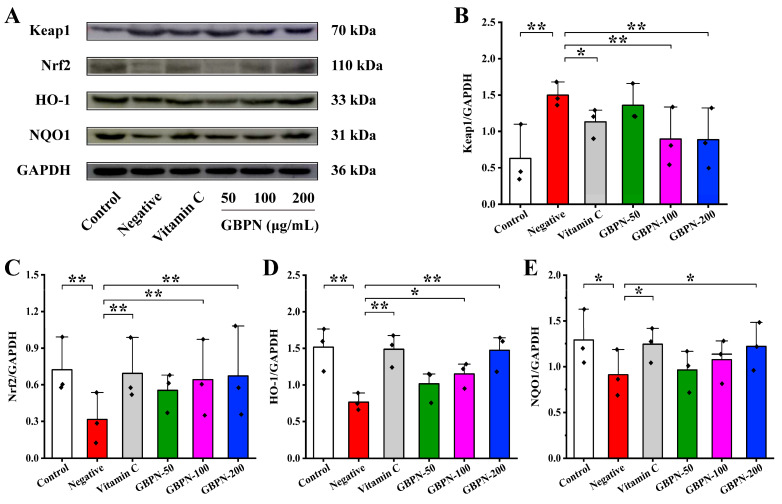
Effects of GBPN on the Keap1/Nrf2/HO-1/NQO1 pathway in D-gal-induced aging-like mice. (**A**) WB bands of Keap1, Nrf2, HO-1, NQO1, and GAPDH. (**B**–**E**) Relative expression levels of Keap1, Nrf2, HO-1 and NQO1. Data are presented as mean ± SD (*n* = 3 independent experiments). Statistical significance was determined by one-way ANOVA followed by Tukey’s post hoc test. * *p* < 0.05, ** *p* < 0.01 vs. Negative group.

In the present study, the expression level of Keap1 was significantly increased, and that of total Nrf2, HO-1 and NQO1 was significantly decreased in the brains of D-gal-induced mice (Figure 7A). GBPN treatment changed the above expression trend, and compared with the Negative group, the expression level of Keap1 was decreased in the GBPN group (Figure 7B), and that of total Nrf2, HO-1 and NQO1 was increased (Figure 7C–E), and the decrease and increase were dose-dependent. The synergistic changes in Keap1, total Nrf2, and their downstream targets HO-1 and NQO1 align with the regulatory mechanism of the Keap1/Nrf2/HO-1/NQO1 antioxidant pathway. Thus, GBPN’s anti-aging effects are presumed to be associated with the Keap1/Nrf2/HO-1/NQO1 pathway.

The structural characteristics of polysaccharides are closely related to their activities, and this study showed that GBPN is a neutral polysaccharide with potential anti-aging properties, which is in agreement with some studies on the anti-aging of neutral polysaccharides. Ji et al. isolated a neutral polysaccharide (AP) with a molecular weight of 34.17 kDa from *Anthriscus sylvestris* (L.) Hoffm, which is composed of Glc, Xyl, Gal, Man and Ara, with a backbone primarily of 1,4-α-D-Glc and minor branching at 1,4,6-α-D-Man. This polysaccharide enhances the activity of antioxidant enzymes in D-gal-induced aged mice via the Nrf2/HO-1/NQO1 pathway [38]. In addition, *Aronia melanocarpa* polysaccharide (AMP) could attenuate oxidative stress damage in the brain tissues of aging mice by modulating the Nrf2/HO-1 pathway, which in turn ameliorated their spatial learning and memory deficits, and AMP consisted of Fuc (0.14%), Rha (0.73%), Ara (7.14%), Gal (10.61%), Glc (76.16%), Xyl (2.31%), Man (1.25%), Gal-UA (1.43%), Glc-UA (0.16%), and Man-UA (0.07%) as neutral polysaccharides [39]. These similarities support the notion that GBPN may exert its anti-aging effects at least partially through interactions with the Keap1/Nrf2/HO-1/NQO1 signaling axis. Compared to the aforementioned neutral polysaccharides, GBPN possesses several unique characteristics. First, it exhibits a relatively low molecular weight (15.2 kDa), for instance, compared to wild celery polysaccharides (34.17 kDa). This lower molecular weight may contribute to enhanced solubility and bioavailability. Second, its unique monosaccharide composition—predominantly glucose (53.18%), arabinose (24.3%), and galactose (16.75%)—differs from previously reported anti-aging polysaccharides AP and AMP, which exhibit distinct monosaccharide compositions and ratios. Third, GBPN adopts a triple-helix conformation, a structural feature associated with enhanced biological activity in numerous polysaccharides. Given this, along with GBPN’s potent antioxidant activity, favorable safety profile, and natural origin, we propose that GBPN holds promise as a dietary supplement or functional food ingredient to support brain health and mitigate age-related cognitive decline.

In summary, our data indicate that GBPN is associated with favorable changes in the Keap1/Nrf2/HO-1/NQO1 pathway in the brains of D-gal-induced aging-like mice. Future studies incorporating nuclear-cytoplasmic fractionation, Nrf2 inhibitors, or genetic models will be valuable to further elucidate the precise mechanistic role of this pathway in GBPN-mediated neuroprotection.

#### 3.4.5. Limitations of the Study

Several limitations of this study must be acknowledged. First, our findings are based on a D-gal-induced aging-like model in male mice. While this established model accelerates age-related phenotypes and is suitable for preliminary efficacy screening, it fails to fully replicate the complexity of natural aging. Second, to control for estrous cycle-related variability, only male animals were used. While this is standard practice for initial pharmacology studies, it precludes the assessment of potential gender differences in GBPN’s anti-aging effects. Future research should include samples from both sexes to determine whether its efficacy is universal or gender-specific. Third, the experimental design lacked a control group of young, healthy mice (without D-gal induction) receiving GBPN treatment alone. Such a control would have effectively distinguished whether long-term GBPN administration itself affects baseline physiological or molecular parameters. Fourth, this study did not perform neurohistological assessments or synaptic marker analyses, which could have provided more direct structural and functional evidence linking the observed cognitive improvements. Finally, the sample sizes across groups were relatively small, reflecting the application of the 3Rs principle in animal research and the preliminary exploratory nature of this study. These limitations underscore the need for future research employing more comprehensive experimental designs: incorporating both sexes, expanding cohort sizes, adding control groups, and conducting multidimensional mechanism analyses to fully elucidate GBPN’s pharmacological characteristics, efficacy, and safety profile.

## 4. Conclusions

In summary, a neutral polysaccharide (GBPN) with a molecular weight of 1.52 × 10^4^ Da was isolated from ginseng berry. Its primary components include Glc, Ara, and Gal, which exist in various glycosidic bond configurations and exhibit a triple-helix structure. GBPN exhibits potent antioxidant activity in vitro, effectively scavenging DPPH radicals and ^•^OH while chelating ferrous ions. In the D-gal-induced aging-like mouse model, GBPN administration significantly alleviated oxidative stress in systemic and brain tissues, manifested as restored antioxidant enzyme (SOD, CAT, GSH-Px) activity and reduced MDA levels. Concurrently, GBPN improved cognitive deficits in aging-like mice, shortening escape latency and enhancing spatial memory in the MWM test. Research indicates these protective effects are mechanistically linked to the activation of the Keap1/Nrf2/HO-1/NQO1 pathway. Our findings highlight GBPN as a promising natural antioxidant agent, warranting further investigation for its potential in mitigating aging-related cognitive decline. It is important to note that these findings are based on a chemically induced aging-like model, and further research is needed to determine their relevance to natural aging or chronic neurodegenerative conditions. Additionally, since polysaccharides often act through multiple pathways, whether GBPN exerts its effects via other distinct antioxidant or stress-response mechanisms warrants further investigation.

## Figures and Tables

**Table 1:** Methylation analysis of GBPN using GC–MS.

Time	PMAAs	Linkage Type	Molar Ratios (%)
13.49	2,3,5-Me_3_-Ara*f*	→1)-Ara*f*	9.73
14.44	2,3,4-Me_3_-Ara*p*	→1)-Ara*p*	5.82
15.73	3,4-Me_2_-Ara*p*	→2)-Ara*p*-(1→	0.66
16.37	2,3-Me_2_-Ara*f*	→5)-Ara*f*-(1→	7.62
17.23	2,3,4,6-Me_4_-Man*p*	→1)-Man*p*	4.86
17.67	2,3,4,6-Me_4_-Gal*p*	→1)-Gal*p*	3.58
18.04	2-Me_1_-Ara*f*	→3,5)-Ara*f*-(1→	1.69
18.27	3-Me_1_-Rha*p*	→2,4)-Rha*p*-(1→	1.34
19.13	2,3,6-Me_3_-Glc*p*	→4)-Glc*p*-(1→	32.95
19.80	2,4,6-Me_3_-Gal*p*	→3)-Gal*p*-(1→	1.59
20.13	2,3,4-Me_3_-Gal*p*	→6)-Glc*p*-(1→	13.81
21.34	2,3-Me_2_-Gal*p*	→4,6)-Glc*p*-(1→	3.70
21.82	2,4-Me_2_-Gal*p*	→3,6)-Gal*p*-(1→	12.67

## Data Availability

For privacy reasons, the original data is not publicly available. However, the datasets used and/or analyzed in the current study are available from the corresponding author upon reasonable request.

## Supplementary Materials

The following supporting information can be downloaded at https://www.mdpi.com/article/10.3390/antiox15010065/s1: Figure S1: Total ion gas chromatogram of GBPN; Figure S2: MS ion fragment diagram of GBPN.

## Abbreviations

The following abbreviations are used in this manuscript:

Ara	Arabinose
CAT	Catalase
D-gal	D-Galactose
DPPH	2,2-Diphenyl-1-picrylhydrazyl
FT-IR	Fourier-Transform Infrared Spectroscopy
Gal	Galactose
GB	Ginseng berry
GBP	Ginseng berry crude polysaccharide
GBPN	Ginseng berry neutral polysaccharide
GC-MS	Gas Chromatography-Mass Spectrometry
Glc	Glucose
GSH-Px	Glutathione peroxidase
HO-1	Heme oxygenase 1
HPGPC	High-performance gel permeation chromatography
HPLC	High-performance liquid chromatography
Keap1	Kelch-like ECH-associated protein 1
MDA	Malondialdehyde
MWM	Morris water maze
NQO1	NAD(P)H: quinone oxidoreductase 1
Nrf2	Nuclear factor erythroid 2-related factor 2
PMP	1-phenyl-3-methyl-5-pyrazolone
PMAAs	Partially methylated alditol acetates
Rha	Rhamnose
SEC-MALLS	Size exclusion chromatography-multi-angle laser light scattering
SEM	Scanning Electron Microscope
SOD	Superoxide dismutase
T-AOC	Total antioxidant capacity
