# Supplementary material for: A Neutral Polysaccharide from Ginseng Berry Mitigates D-Galactose-Induced Oxidative Stress and Cognitive Deficits Through the Keap1/Nrf2/HO-1/NQO1 Pathway
Source: Antioxidants (Basel). 2026 Jan 3;15(1):65. doi: 10.3390/antiox15010065 (PMC12837864; doi:10.3390/antiox15010065)
Supplement: Supplementary file 1 [file antioxidants-15-00065-s001.zip › antioxidants-4006077-supplementary.pdf]

Supplementary material

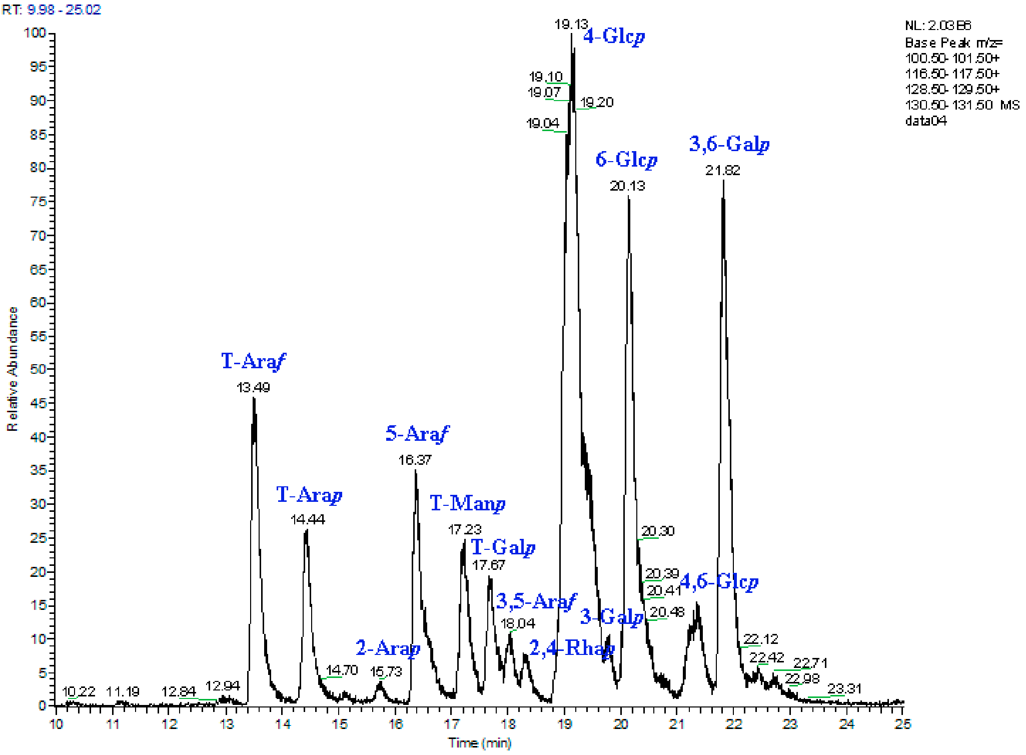

Figure S1. Total ion gas chromatogram of GBPN.

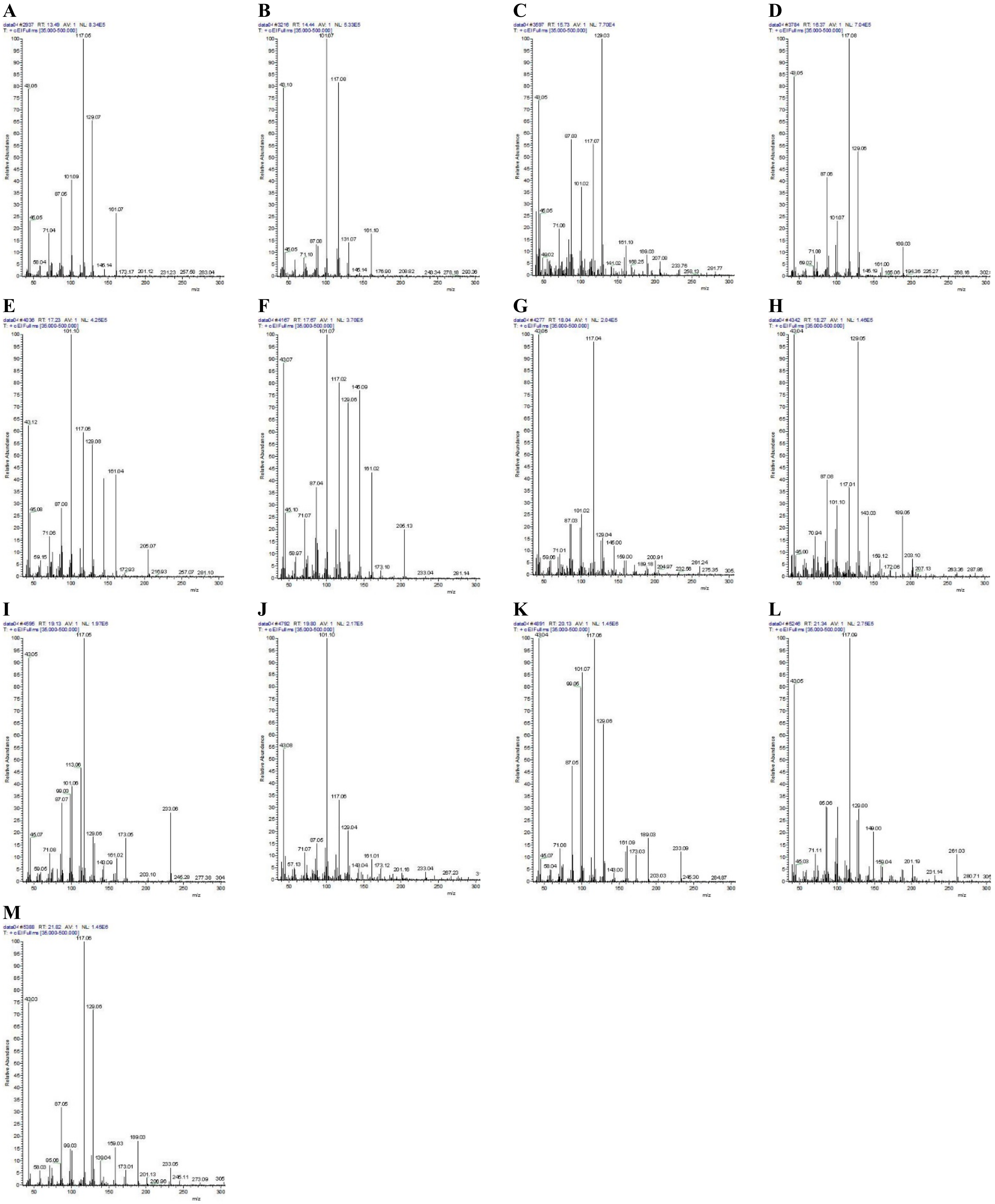

Figure S2. MS ion fragment diagram of GBPN.
